# Stability and Hydrocarbon/Fluorocarbon Sorption of a Metal-Organic Framework with Fluorinated Channels

**DOI:** 10.3390/ma9050327

**Published:** 2016-04-29

**Authors:** Jijiang Xie, Fuxing Sun, Chunrui Wang, Qikun Pan

**Affiliations:** 1 State Key Laboratory of Laser Interaction with Matter, Changchun Institute of Optics, Fine Mechanics and Physics, Changchun 130033, China; laserxjj@163.com (J.X.); crwang@ciomp.ac.cn (C.W.); panqikun2005@163.com (Q.P.); 2 Innovation Laboratory of Electro-Optical Countermeasures Technology, Changchun Institute of Optics, Fine Mechanics and Physics, Changchun 130033, China; 3 State Key Laboratory of Inorganic Synthesis and Preparative Chemistry, Jilin University, Changchun 130012, China

**Keywords:** fluorocarbon sorption, microporous materials, hydrothermal stability

## Abstract

The stabilities and hydrocarbon/fluorocarbon sorption properties of a zeolite-like metal-organic framework (MOF) Zn(hfipbb) with fluorinated channels has been studied. By the combination of thermogravimetric analysis (TGA) and powder X-ray diffraction (PXRD) measurements, we confirm that Zn(hfipbb) has exceptionally high hydrothermal and thermal stabilities. The adsorption behaviors of water and methanol by Zn(hfipbb) indicate that it is highly hydrophobic but with high adsorption of alcohols. Hexane and perfluorohexane adsorption measurements show that the fluorinated channels in Zn(hfipbb) have high affinity with hydrocarbon and fluorocarbon. The high fluorophilic nature of the channels and the high stability of the compound suggest its potential utility in practical separation applications.

## 1. Introduction

Metal-organic frameworks (MOFs) are the novel kind of crystalline microporous materials that are constructed from metal ions or metal clusters and organic linkers by coordination bonds [1]. Owing to their high and tunable structural and porous properties, they have attracted wide interest in recent years in many functional applications in terms of gas sorption and separations, catalysis, sensors, biomedicine, *etc.* [2]. On the other hand, to explore microporous materials with advanced gas sorption and separation properties requires an understanding of the physical adsorption and desorption of guest molecules onto the surface of a solid [3,4]. Microporous MOFs are the ideal investigated subjects because of their structural certainty and tunable inner surface.

Hydrocarbons are very important raw chemicals and energy resources. The sorption and separation of hydrocarbons is a big and challenging issue in the petroleum industry. Fluorocarbon and its derivatives are also of great practical and industrial interest because of their usefulness in many applications, such as used as refrigerants and propellants, and in the electronics industry [5]. However, many MOFs with high porosity are not water stable and thus cannot survive long after exposure to humid air, because water or other nucleophiles would attack the metal-containing clusters and substitute the ligands [6]. The instabilities become a critical issue of the practical uses of many MOFs. The incorporation of fluorine atoms in the channels of MOF will lead to the hydrophobicity, enhancing the stabilities of the frameworks in water [7,8,9,10,11]. In addition, fluoridated surfaces are expected to improve the adsorption, separation, and catalytic activity of organic fluorinated molecules due to their fluorophilic character. In 2007, Omary and coworkers firstly reported the fluorous MOF, FMOF-1, which shows high gas adsorption and hydrophobicity [12]. Recently, Miljanic and coworkers reported several fluorinated MOFs including two of them with mesopores and showed their superhydrophobic properties and exceptionally high adsorption of fluorocarbons and chlorofluorocarbons (CFCs) [13,14]. Up to now, only a few porous fluorous MOFs have been reported because of the paucity of available fluorinated ligands, the difficult synthesis process, and high cost [15,16].

In this work, we focus on the stability and hydrocarbon/fluorocarbon sorption properties of a zeolite-like MOF, Zn(hfipbb) (H_2_hfipbb = 4,4’-(hexafluoroisopropylidene)bis(benzoic acid), C_17_H_10_F_6_O_4_) with fluorinated channels. H_2_hfipbb is commercially available and is almost the most-used fluorinated ligand in the construction of MOFs [17,18,19]. It is shown that Zn(hfipbb) has exceptionally high hydrothermal and thermal stabilities. The adsorption behavior of Zn(hfipbb) indicates that it is highly hydrophobic but with high affinity to alcohol, fluorocarbon and hydrocarbon.

## 2. Experimental Section

All reagents were commercially available and used as received without further purification. H_2_hfipbb was provided by Accelerating Scientific and Industrial Development thereby Serving Humanity, Beijing, China. Zn(NO_3_)_2_·6H_2_O and isopropanol were provided by Beijing Chemical Works (Beijing, China). Elemental analyses (C, H, and N) were carried out on a Perkin-Elmer 240 analyzer (PerkinElmer, Waltham, MA, USA). Fourier Transform Infrared Spectoscopy (FT-IR) spectra were recorded as KBr pellets with a Nicolet Impact 410 FT-IR spectrometer (Thermo Nicolet Corporation, Madison, WA, USA), using the reflectance technique (4000–400 cm^−1^). Thermogravimetric analysis (TGA) curves were collected on a Perkin-Elmer TGA 7 thermogravimetric analyzer (PerkinElmer, Waltham, MA, USA) with a heating rate of 10 °C/min in air atmosphere. Powder X-ray diffractions (PXRD) were carried out on a Scintag X1 diffractometer (Thermo Electron Corporation, Waltham, MA, USA) with Cu Kα (λ = 1.5418 Å) at 40 kV, 35 mA. Low pressure and high pressure gas adsorption measurements were performed on a Micromeritics ASAP 2010 instrument (Micromeritics instrument (Shanghai) Ltd., Shanghai, China) and a Rubotherm instrument (Rubotherm, Friedrichshafen, Germany) with magnetic suspension balance, respectively. Vapor sorptions were measured on an Ankersmid SGA-100 gas adsorption instrument (Ankersmid, Nijverdal, The Netherlands). All the samples for the sorption measurement were firstly activated by heat at 350 °C in air for 4 h.

In a typical synthesis process of Zn(hfipbb), the mixture of Zn(NO_3_)_2_·6H_2_O (0.06 g, 0.20 mmol) and H_2_hfipbb (0.08 g, 0.20 mmol) in 10 mL H_2_O and 2 mL of isopropanol was heated at 180 °C for three days. Upon cooling to room temperature, colorless, hexagonal rod-shaped crystals were collected by filtration, washed with distilled water and isopropanol, and dried in air to afford Zn(hfipbb) in 87% yield. IR (KBr pellet): 3428 (br), 2967 (w), 2360 (m), 1637 (s), 1540 (s), 1421 (s), 1240 (s), 1176 (s), 931 (m), 725 (s), 517 (m) cm^−1^. Anal. Calcd for C_17_H_8_F_6_O_4_Zn: C, 44.82; H, 1.77; N, 0. Found: C, 44.58; H, 1.63; N, 0.12.

## 3. Results and Discussion

**Figure 1:**
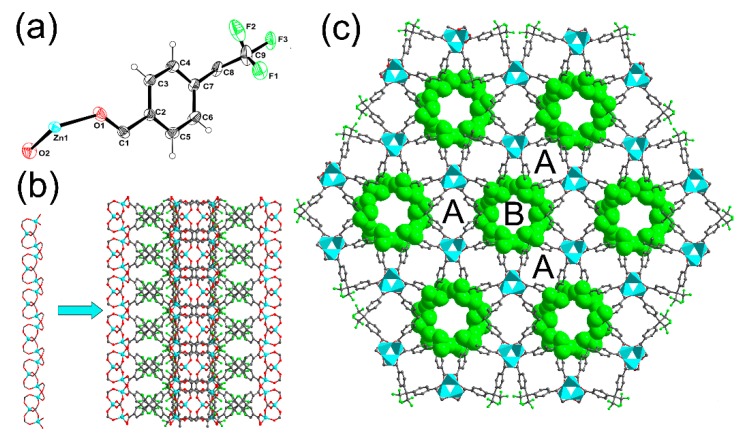
Crystal structure of Zn(hfipbb): (**a**) asymmetric unit (at 50% probability); (**b**) helical chain and the framework view along [100] direction; (**c**) the framework viewed along [001] direction showing channels **A** and **B** (Zn: turquoise polyhedral; F: green sphere).

The structure and synthesis of Zn(hfipbb) was firstly reported by Monge and coworkers [20]. They synthesized the sample by the hydrothermal reactions of the mixture Zn(NO_3_)_2_·6H_2_O and the ligand H_2_hfipbb at 170 °C for three days. However, because the ligand H_2_hfipbb is highly hydrophobic, the ligand would be very difficult if fully dispersed in the water, which results in the low yield of the product. Therefore, we improve the synthesis process by adding isopropanol to disperse the ligand and raise the yield. The structure of Zn(hfipbb) is a three-dimensional framework, crystallized in the high-symmetry chiral space group *P*6_4_22. The asymmetrical unit of this compound contains one half tetrahedral Zn(II) ion and one half of the ligand hfipbb (see Figure 1a), thus the composition was found to be Zn(C_17_H_8_F_6_O_4_). The Zn(II) nodes are bridged by the carboxylic groups to generate a 3_1_ helical chains along *c* directions. Each helical chain connects to three identical adjacent chains through the ligands hfipbb, resulting in a 3D framework (see Figure 1b). The whole framework contains two types of open channels running along the *c* direction (see Figure 1c). Channel **A** lies on a two-fold symmetry axis and has small windows (circa 3.8 × 3.8 Å^2^, excluding the van der Waals radius of carbon) to larger cages (circa 6.0 × 6.0 Å^2^) at intervals of 7.7 Å (the length of the large cage), which is similar to the channels previously reported by Li and co-workers [17]. Channel **B** lies on a 6_4_ screw axis and is homochiral. The CF_3_ groups of the hfipbb^2−^ ligands protrude into this channel from the sides of the hexagonal windows (circa 6.5 Å in diameter), affording a fluoridated internal surface. The void volume, as calculated using PLATON [21], is 25.3% of the total volume.

**Figure 2:**
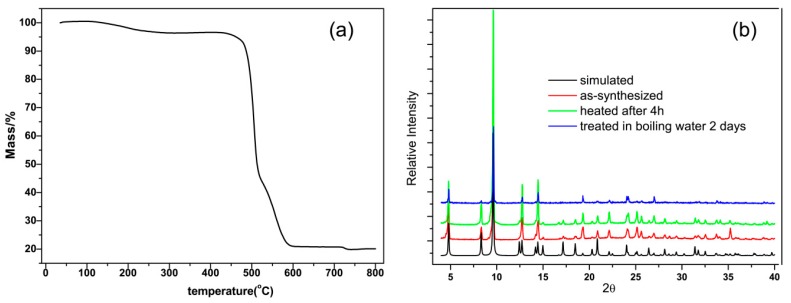
(**a**) TGA curve of Zn(hfipbb) in air; (**b**) PXRD patterns of Zn(hfipbb).

The thermogravimetric analysis (TGA) of the as-synthesized Zn(hfipbb) under aerobic conditions revealed the compound to be compositionally robust up to 450 °C, only with a small weight loss of 4%, indicating a few guest molecules in the channel (see Figure 2a). The thermal stabilities of Zn(hfipbb) were confirmed by powder X-ray diffraction (PXRD). As shown in Figure 2b, a sample can be heated to 400 °C in air for 4 h without loss of crystallinity. However, the framework would be decomposed after being heated to 400 °C in air for 6 h (see Figure S1). In addition, the hydrothermal stability was examined by suspending a sample in boiling water. After two days, the sample had retained its main crystallinity, as also verified by PXRD. These results demonstrate that Zn(hfipbb) may be suitable for applications that require frequent adsorption-desorption cycles over long periods of time, and for catalysis under hydrothermal conditions.

**Figure 3:**
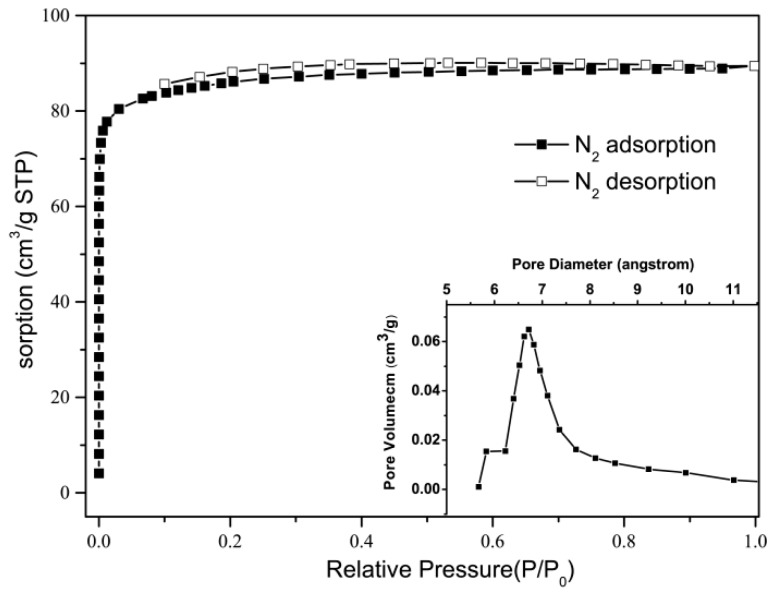
N_2_ sorption isotherms of Zn(hfipbb) at 77 K (insert: the pore width distribution).

The microporous feature of Zn(hfipbb) was confirmed by N_2_ sorption measurement at 77 K. The sample was firstly activated by heat at 350 °C in air for 4 h. The observed N_2_ sorption isotherm revealed a typical Type I adsorption curve and a Brunauer-Emmett-Teller (BET) surface area of 287 m^2^/g (see Figure 3). Using the *t*-plot model, the pore volume is estimated as 0.112 cm^3^/g, which is smaller than 0.187 cm^3^/g calculated from the crystal structure. This discrepancy can be attributed to hindrance of the N_2_ molecules entering the channels and not filling the void space efficiently. Using the Horvath-Kawazoe (HK) method, only one narrow pore width distribution centered at 6.7 Å was obtained, which is consistent with the diameter of channel **B** and proves that channel **A** is too small for N_2_ to enter in. Both H_2_ and CH_4_ adsorption isotherms were also measured and found to exhibit Type-I behavior (see Figures S2 and S3). The gravimetric density of 1.4 wt % of CH_4_ was achieved at 298 K and 40 bar, corresponding to 29 V(STP)/V. The excess H_2_ uptake in Zn(hfipbb) is 1.1 wt % at 77 K and 80 bar, however, only 0.12 wt % H_2_ can be adsorbed at 298 K and 80 bar. Owing to its low porosity and surface area, the H_2_ and CH_4_ adsorption ability of Zn(hfipbb) is lower than the record of other MOFs for these applications [22,23]. These results indicate that Zn(hfipbb) is not proper for the storage of these gases; however, the high stabilities as well as the small and uniform pores of Zn(hfipbb) would allow them to be used for gas separation under more severe conditions.

**Figure 4:**
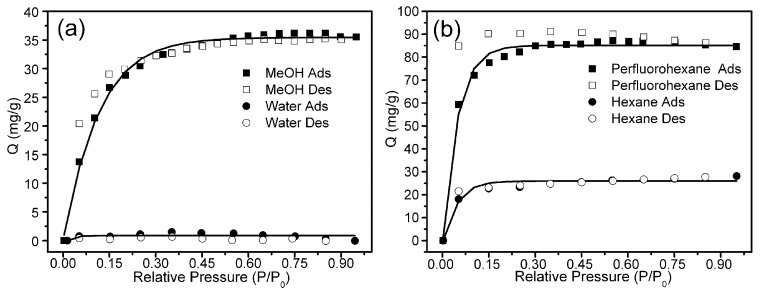
Sorption isotherms of Zn(hfipbb) at room temperature: (**a**) water and methanol; (**b**) hexane and perfluorohexane.

The nature of the pores in Zn(hfipbb) allows for a variety of small molecules to be adsorbed. In order to study the pore features of Zn(hfipbb), we measured the sorption isotherms of some solvent vapors by the activated sample of Zn(hfipbb) at room temperature. The sorption measurement of water by Zn(hfipbb) shows that the compound was fully hydrophobic, exhibiting essentially zero water adsorption (see Figure 4a). These results indicate that the two channels in Zn(hfipbb) are both hydrophobic, which is not as Monge and coworkers reported [20]. In contrast, it adsorbs up to 36 mg/g of methanol displaying a Type I isotherm.

Hexane and perfluorohexane adsorption isotherms were also measured in order to probe the influence of the fluorinated channels. As the length of these molecules is longer than the interval distance of its large cages, they should not be adsorbed within channel **A** [17]. In addition, perfluorohexane is widely used in the electronic cooling liquid/insulator for the low-temperature applications. In addition, hexanes, the homologue of perfluorohexane, are significant constituents of gasoline and widely used as cheap, relatively safe, largely unreactive, and easily evaporated non-polar solvents. Figure 4b shows the hexane and perfluorohexane adsorption isotherms which are both typical Type I. At saturation, Zn(hfipbb) adsorbs 28 mg/g of hexane and 87 mg/g of perfluorohexane, which corresponds to 0.8 and 0.63 guest molecules per unit cell. The adsorptive capacity is not high compared to other reported MOFs, although the MOFs are very few [13,24]. The sorption behaviors by Zn(hfipbb) indicate that the fluorinated channels **B** have high affinity to fluorocarbon and hydrocarbon. In the low *P*/*P*_0_ region, the adsorption of these vapors reached saturation indicating strong interactions between the guest and host. In addition, an obvious hysteresis phenomenon could be observed in the sorption of perfluorohexane by Zn(hfipbb), and the desorption can not be accomplished by vacuum. These results demonstrate that the fluorinated channel **B** Zn(hfipbb) has a high fluorophilic character. The fluorophilic features of Zn(hfipbb) promise that it can be used for selective adsorption of hydrocarbon or fluorocarbon, particularly under high-humidity conditions.

## 4. Conclusions

In conclusion, we reported the stabilities and hydrocarbon/fluorocarbon sorption properties of a zeolite-like MOF Zn(hfipbb) with fluorinated channels. By the combination of TGA and PXRD measurements, we confirm that Zn(hfipbb) has exceptionally high hydrothermal and thermal stabilities. The adsorption behaviors of water and methanol by Zn(hfipbb) indicate that it is highly hydrophobic but with high adsorption of alcohols. Hexane and perfluorohexane adsorption measurements show that the fluorinated channels are with high affinity to hydrocarbon and fluorocarbon. The high fluorophilic nature of the channels and the high stability of the compound suggest its potential utility in practical separation applications.

## Supplementary Materials

The following are available online at www.mdpi.com/1996-1944/9/5/327/s1. Figure S1: PXRD patterns of Zn(hfipbb) after heated to 400 °C in air; Figure S2: Hydrogen adsorption isotherms of Zn(hfipbb) at 77 K and 298 K; Figure S3: Methane adsorption isotherm of Zn(hfipbb) at 298 K.
