# Supplementary material for: Stability and Hydrocarbon/Fluorocarbon Sorption of a Metal-Organic Framework with Fluorinated Channels
Source: Materials (Basel). 2016 Apr 29;9(5):327. doi: 10.3390/ma9050327 (PMC5503081; doi:10.3390/ma9050327)
Supplement: Supplementary file 1 [file materials-09-00327-s001.pdf]

Supplementary Materials: Stability and Hydrocarbon/Fluorocarbon Sorption of a Metal-Organic Framework with Fluorinated Channels

Jijiang Xie, Fuxing Sun, Chunrui Wang and Qikun Pan

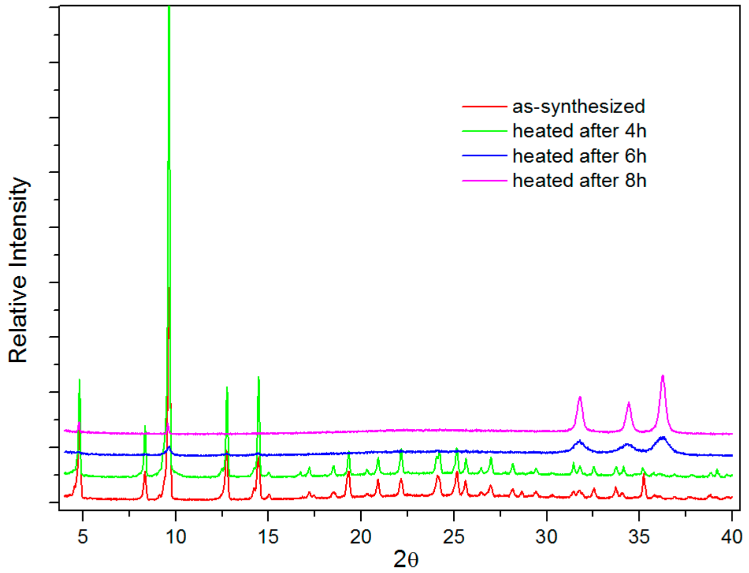

Figure S1. PXRD patterns of Zn(hfipbb) after heated to 400 °C in air.

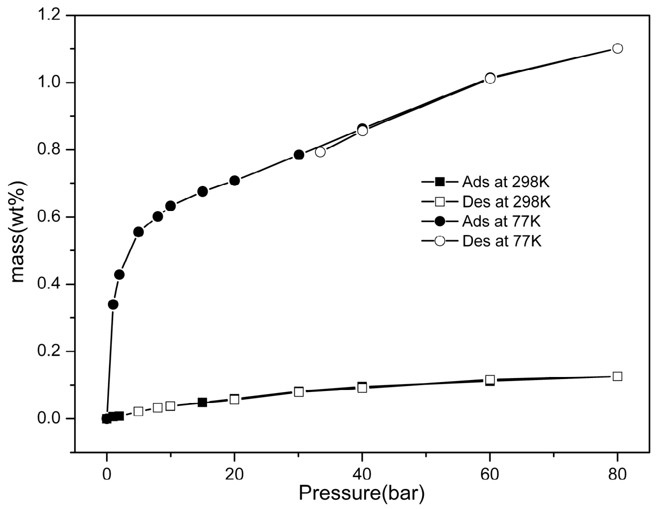

Figure S2. Hydrogen adsorption isotherms of Zn(hfipbb) at 77 K and 298 K.

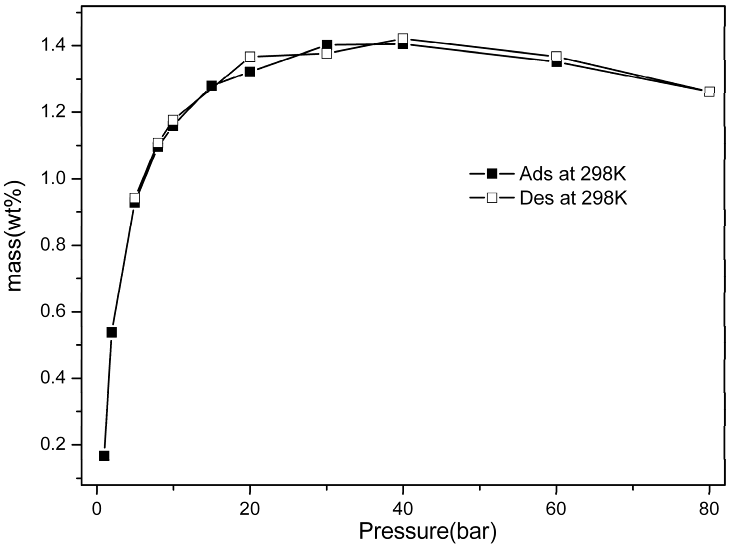

Figure S3. Methane adsorption isotherm of Zn(hfipbb) at 298 K.
